# Epigenetic Control of Virulence Gene Expression in *Pseudomonas aeruginosa* by a LysR-Type Transcription Regulator

**DOI:** 10.1371/journal.pgen.1000779

**Published:** 2009-12-18

**Authors:** Keith H. Turner, Isabelle Vallet-Gely, Simon L. Dove

**Affiliations:** Division of Infectious Diseases, Children's Hospital, Harvard Medical School, Boston, Massachusetts, United States of America; Universidad de Sevilla, Spain

## Abstract

Phenotypic variation within an isogenic bacterial population is thought to ensure the survival of a subset of cells in adverse conditions. The opportunistic pathogen *Pseudomonas aeruginosa* variably expresses several phenotypes, including antibiotic resistance, biofilm formation, and the production of CupA fimbriae. Here we describe a previously unidentified bistable switch in *P*. *aeruginosa*. This switch controls the expression of a diverse set of genes, including *aprA*, which encodes the secreted virulence factor alkaline protease. We present evidence that bistable expression of PA2432, herein named *bexR* (bistable expression regulator), which encodes a LysR-type transcription regulator, controls this switch. In particular, using DNA microarrays, quantitative RT–PCR analysis, chromatin immunoprecipitation, and reporter gene fusions, we identify genes directly under the control of BexR and show that these genes are bistably expressed. Furthermore, we show that *bexR* is itself bistably expressed and positively autoregulated. Finally, using single-cell analyses of a *GFP* reporter fusion, we present evidence that positive autoregulation of *bexR* is necessary for bistable expression of the BexR regulon. Our findings suggest that a positive feedback loop involving a LysR-type transcription regulator serves as the basis for an epigenetic switch that controls virulence gene expression in *P*. *aeruginosa*.

## Author Summary

Bistable switches allow the expression of a gene, or set of genes, to switch from one stable expression state to another and can generate cells with different phenotypes in an isogenic population. In this work we uncover a previously unidentified bistable switch that controls virulence gene expression in the opportunistic pathogen *P*. *aeruginosa*. This switch is controlled by a LysR-type transcription regulator that we call BexR. As well as identifying specific genes that are regulated by BexR, we show that *bexR* is itself bistably expressed and positively autoregulated. Furthermore, we present evidence that positive autoregulation of *bexR* is necessary for bistable expression of the BexR regulon. Our findings support a model for BexR-mediated bistability in which positive feedback amplifies *bexR* expression in a stochastically determined subset of cells, giving rise to heterogeneous expression of BexR target genes within a cell population. By generating diversity in an isogenic population of *P*. *aeruginosa* this bistable switch may ensure the survival of a subset of cells in adverse conditions, such as those encountered in the host. Our study defines an epigenetic mechanism for phenotypic variation in *P*. *aeruginosa*.

## Introduction

The Gram-negative bacterium *Pseudomonas aeruginosa* is an opportunistic pathogen of humans. It can cause infection in a wide variety of tissues in the immunocompromised host, and is the leading cause of morbidity and mortality in cystic fibrosis (CF) patients [1]. This breadth of infectious capacity is thought to result from differential gene expression, as genomic variability between clinical and environmental isolates is low and the genome of *P*. *aeruginosa* encodes a high proportion of transcription regulators [2],[3]. Studying the mechanisms and outcomes of transcription regulation in *P*. *aeruginosa* may offer some insight into how cohorts of virulence factors are coordinately expressed to influence pathogenesis in a range of pseudomonal infections.

Bacteria are traditionally thought to use transcription regulation to adapt to changing environmental conditions, such as the presence of a new carbon or energy source, a change in temperature or pH, or introduction to a host environment. However, in harsh environmental conditions that exert a sudden selective pressure on a population of cells, the time needed to respond using a genetic regulatory network may prove fatal. The ability of isogenic populations of bacteria to exhibit phenotypic variation allows them to cope with such situations by pre-adapting a subset of the population to the sudden introduction of harsh conditions. Several examples of phenotypic variation in *P*. *aeruginosa* have been identified, such as the phase-variable expression of the *cupA* fimbrial gene cluster under anaerobic conditions and the transient formation of antibiotic resistant, hyperadherent rough small-colony variants under antibiotic exposure [4]–[7]. These phenotypes may contribute to the ability of infecting bacteria to withstand chemical or mechanical insults encountered during colonization of the CF lung. Examples such as these suggest that phenotypic variation by *P*. *aeruginosa* allows the organism to thrive in a complex environment. However, the mechanisms by which these phenotypes are variably expressed are unknown.

Phenotypic variation in bacteria can arise from a variety of mechanisms, both genetic and epigenetic in nature. Classical phase-variation is thought to be genetically mediated, such as the variable expression of the flagellum in *Salmonella enterica* serovar Typhimurium, which is mediated by specifically catalyzed changes in promoter DNA orientation [8]. Phase-variation can also be mediated by epigenetic mechanisms, such as the one involving DNA methylation that controls the phase-variable expression of pyelonephritis-associated pili genes in uropathogenic *Escherichia coli* [9],[10]. Phenotypic heterogeneity can arise in the absence of DNA sequence variation or DNA modification in bistable systems (i.e. systems that can exist in one of two alternative expression states, and reversibly switch between them), such as in the case of the lysogenic switch of bacteriophage λ [11],[12]. Bistability can arise when there exists a mechanism for amplifying differences in protein levels between individual cells and stably propagating these differences to daughter cells (reviewed in [13]). The bistable expression of genes can be achieved using a positive regulatory feedback loop, as is the case in the development of competence under nutrient limitation in *Bacillus subtilis*; positive feedback of ComK, the master regulator of competence, is required for bistable development of competence in *B*. *subtilis* [14],[15]. Thus, the architecture of a particular gene regulatory circuit can enable stochastic, reversible differentiation of subsets of bacterial populations into distinct cell types.

Here we uncover a previously unidentified bistable switch in *P*. *aeruginosa* controlled by BexR, a LysR-type transcription regulator. We demonstrate that *bexR* is itself bistably expressed in a BexR-dependent manner and that BexR positively regulates the expression of its own gene. Using DNA microarrays and quantitative real-time RT-PCR (qRT-PCR), we define the bistable regulon of BexR, which contains a diverse set of genes and includes *aprA*, which encodes the virulence factor alkaline protease. We show further that BexR acts directly at the promoters of many of its regulatory targets, including that of its own gene. Finally, we describe a series of single-cell population analyses that suggest that this bistable switch requires *bexR* autoregulation. We propose a model for the BexR switch in which positive feedback amplifies *bexR* expression in a stochastically determined subset of cells, giving rise to bistable expression of BexR target genes in an isogenic population.

## Results

### BexR Is a Positive Regulator of PA1202 Bistability

**Figure 1:**
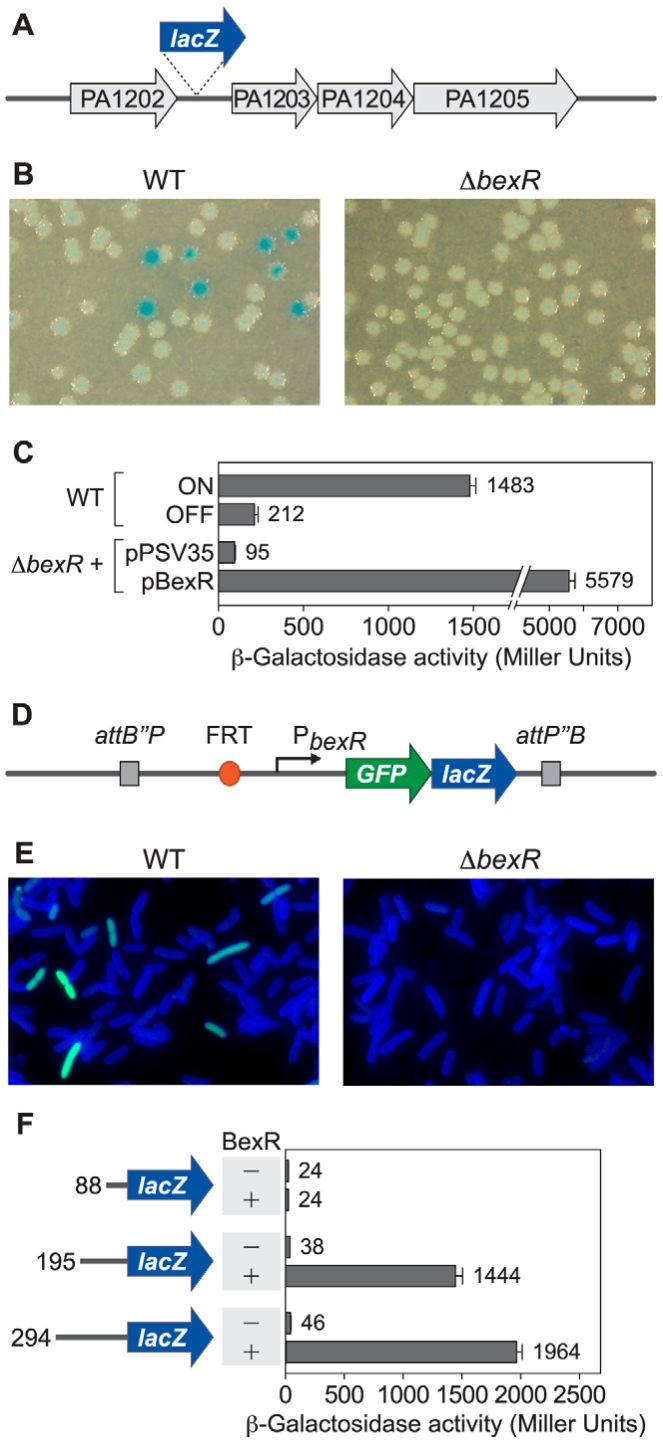
BexR is a positively autoregulated, bistably expressed regulator of PA1202. (A) Schematic of *PA1202 lacZ* reporter strains. (B) Phenotypes of wild-type and Δ*bexR PA1202 lacZ* reporter strains when plated on LB agar containing X-Gal. (C) Quantification of PA1202 *lacZ* expression in cultures of the wild-type reporter strain in both ON and OFF states, and the Δ*bexR* reporter strain with empty vector and *bexR* expression vector. (D) Schematic of *attB::P_bexR_-GFP-lacZ* reporter strain. (E) Micrographs of wild-type and Δ*bexR attB::P_bexR_-GFP-lacZ* fluorescent reporter cells stained with the membrane dye TMA-DPH. (F) Quantification of *P_bexR_-lacZ* expression in PAO1 Δ*bexR* using varying lengths of *bexR* promoter DNA in cells with empty vector and *bexR* expression vector. Error bars in (C,F) represent 1 SD from the mean *β*-galactosidase activity.

In the course of unrelated microarray experiments, we observed a small set of genes that exhibited variable expression between replicates of wild-type cultures of *P*. *aeruginosa* strain PAO1 (data not shown). This set includes PA1202, which encodes a hypothetical protein with homology to a predicted hydrolase of *Escherichia coli*, and PA2432 (herein named *bexR* for *b*istable *ex*pression *r*egulator), which is predicted to encode a member of the LysR family of transcription regulators. To confirm that PA1202 is expressed in a variable manner, we constructed a strain of PAO1 in which *lacZ* was placed downstream of the PA1202 gene (Figure 1A). This strain exhibits reversible bistable expression of the *lacZ* reporter. Specifically, wild-type cells of this reporter strain give rise to both blue (“ON”) and white (“OFF”) colonies on LB agar plates containing X-Gal (Figure 1B). When re-streaked on LB agar with X-Gal, ON colonies give rise to both ON and OFF colonies, and OFF colonies give rise to both OFF and ON colonies. Because our initial microarray analyses suggested that *bexR*, which encodes a putative transcription activator, co-varied with PA1202, we hypothesized that BexR may positively regulate expression of PA1202 and that bistable expression of *bexR* may be responsible for the observed bistability in PA1202 expression. To begin to test this hypothesis, we constructed an unmarked in-frame deletion of *bexR* in PAO1 *PA1202 lacZ*. Compared to the wild-type reporter strain, the Δ*bexR* mutant exhibits constitutively low-level expression of PA1202 (Figure 1B). Ectopic expression of *bexR* in the Δ*bexR* mutant resulted in increased PA1202 expression (Figure 1C), suggesting that BexR positively regulates expression of PA1202. However, bistable expression of PA1202 is lost when *bexR* is expressed ectopically; PAO1 Δ*bexR PA1202 lacZ* grows only as ON colonies on LB agar with X-Gal when carrying a plasmid containing *bexR* (data not shown), suggesting that native regulation of *bexR* is necessary for bistable PA1202 expression. Quantification of the frequency at which this switch in expression state occurs reveals a relatively infrequent switch with a bias in favor of the OFF to ON transition (Table 1).

**Table 1:** Switching frequencies.

Strain Genotype	Transition	Switching Frequency (per cell generation, x10^−4^)
*PA1202 lacZ*	OFF → ON	47.8±3.0
	ON → OFF	0.8±0.2
Δ*bexR PA1202 lacZ*	OFF → ON	<0.4±0.1

### The *bexR* Gene Is Bistably Expressed and Positively Autoregulated

To determine whether *bexR*, like PA1202, is expressed in a bistable manner, we constructed a reporter strain in which the putative *bexR* promoter region was placed upstream of a *GFP*-*lacZ* reporter in single copy at the ΦCTX attachment site in the PAO1 chromosome (Figure 1D) [16],[17]. Individual cells of wild-type PAO1 carrying this *P_bexR_*-*GFP*-*lacZ* reporter either express the GFP reporter, or do not, leading to heterogeneity in the cell population (Figure 1E). Interestingly, cells lacking BexR exhibit constitutively low-level expression of the reporter, suggesting that bistable expression from the *bexR* promoter also depends on BexR. Bistable expression from the *bexR* promoter was also observed at the colony level, suggesting long-term maintenance of the BexR expression state (Figure S1). The frequency of switching between expression states is similar for *bexR* and PA1202, further supporting the hypothesis that bistable expression of *bexR* is upstream of PA1202 bistability (Table 1 and Table S1). Truncation of the *bexR* upstream sequence indicated that a 195 bp fragment of upstream DNA is still sufficient to drive expression of a *lacZ* reporter (integrated in single copy in the chromosome) when *bexR* is expressed from a plasmid, whereas an 88 bp fragment is not (Figure 1F). Thus, the 195 bp region of DNA immediately upstream of *bexR* presumably contains the *bexR* promoter and BexR binding site(s). Thus, BexR positively regulates expression of PA1202 and of its own gene, and *bexR* is itself bistably expressed, suggesting that other BexR target genes may also be expressed in a bistable manner.

### BexR Regulates Expression of a Diverse Set of Genes, Including That Encoding the Virulence Factor AprA

To determine the full extent of the BexR regulon in PAO1, we compared the mRNA content of PAO1 Δ*bexR* cells containing either a *bexR* expression vector or an empty vector in both mid-logarithmic and stationary phases of growth using DNA microarrays. A total of 71 genes exhibited between a 2- and 70-fold change in expression, with most genes upregulated by ectopic expression of *bexR* (Figure S2). PA1202 was upregulated 70-fold upon ectopic expression of *bexR* in mid-logarithmic phase. Several genes downstream of PA1202 were also strongly upregulated by ectopic expression of *bexR*, suggesting that these comprise a BexR-regulated operon. This putative operon includes PA1203, which is predicted to encode a redox protein, PA1204, which is predicted to encode a NADPH-dependent FMN reductase, and PA1205, which is predicted to encode a homolog of pirin, a widely conserved protein with oxygenase activity [18]. PA2698, which is also predicted to encode a hydrolase, was upregulated 7-fold by ectopic expression of *bexR*, suggesting that a cohort of several enzymes are coordinately regulated by BexR. Several multidrug efflux pumps appeared to be regulatory targets of BexR, as downregulation of *mexEF*-*oprN* by 6- to 10-fold and upregulation of *mexGHI*-*opmD* by 7- to 13-fold was observed during ectopic expression of *bexR*. Several quorum sensing-regulated genes encoding secreted proteins were also positively regulated by ectopic *bexR* expression, such as PA0572, which encodes a LasR-regulated Xcp secretion substrate with a predicted Zn-metalloprotease motif [19]–[21]. Finally, the LasR-regulated genes *aprX*, *aprE*, *aprF* and *aprA*, which encode components of the alkaline protease production and secretion machinery, were positively regulated by BexR. *aprA*, which encodes the alkaline protease precursor protein, plays a role in virulence in a *Drosophila melanogaster* orogastric model of pseudomonal infection, where it is thought to protect *P*. *entomophila* from antimicrobial peptides [22]. These results suggest that BexR controls the expression of a diverse set of genes, including some that encode predicted enzymes and others that encode quorum-regulated secreted proteases.

### The BexR Regulon

**Figure 2:**
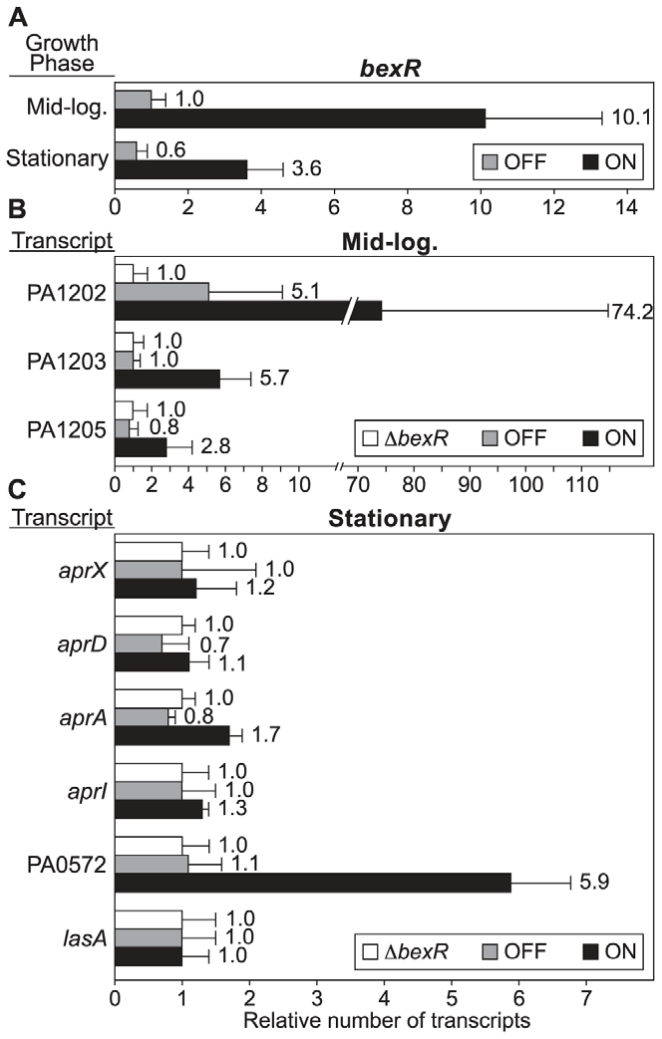
BexR-regulated transcripts vary between the OFF and ON states. (A) Relative quantity of *bexR* mRNA in both mid-logarithmic and stationary phase. (B) Relative quantities of PA1202 operon mRNAs in mid-logarithmic phase. (C) Relative quantities of quorum sensing-regulated mRNAs in stationary phase. Error bars represent relative expression values calculated from +/−1 SD from the mean ΔΔCt.

Because *bexR* is itself bistably expressed we would predict that the expression of BexR target genes in wild-type cells should co-vary with the *bexR* expression state. To test this prediction, we isolated mRNA from cultures of wild-type *attB*::*P_bexR_*-*lacZ* OFF, *attB*::*P_bexR_*-*lacZ* ON and Δ*bexR attB*::*P_bexR_*-*lacZ* reporter strains at both mid-logarithmic and stationary growth phases and profiled relative transcript abundance by qRT-PCR. We observed an approximately 10-fold difference in abundance of *bexR* transcripts between OFF and ON cultures in mid-logarithmic phase, and an approximately 6-fold difference between OFF and ON cultures in stationary phase (Figure 2A). Consistent with the idea that BexR target genes are expressed in a bistable manner in wild-type cells, expression of members of the putative PA1202 operon, from PA1202 to PA1205, all co-varied with *bexR* expression (Figure 2B), as did PA0572 and *aprA* (though for *aprA* the difference in transcript abundance between ON and OFF cultures was only 2-fold) (Figure 2C). We were unable to observe significant bistable expression of the other *apr* genes, possibly due to the relatively modest effect of BexR on their expression. The abundance of the *lasA* transcript was not significantly different between Δ*bexR* and wild-type cultures, suggesting that the observed bistability of *aprA* and PA0572 (which, like *lasA*, are LasR-regulated [19]) is not due to differences in LasR function between ON and OFF cultures (Figure 2C). Microarray analysis of cells ectopically expressing *bexR* suggests that two operons encoding multidrug efflux pumps are reciprocally regulated by BexR (Figure S2). However, this was not observed in wild-type cells in the OFF and ON states (data not shown). Taken together, our data indicate that BexR is responsible for coordinate bistable expression of a variety of genes in wild-type *P*. *aeruginosa*, including two that encode quorum sensing-regulated secreted proteases (PA0572 and *aprA*).

### BexR Acts Directly at Target Promoters

**Figure 3:**
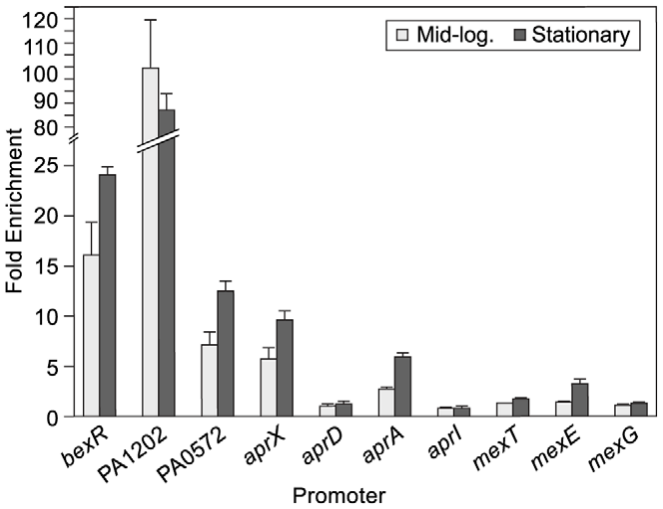
BexR occupies the promoters of target genes. ChIP of an epitope-tagged version of BexR reveals preferential binding of the promoters of target genes over a non-binding control region *in vivo* at both mid-logarithmic and stationary phase. Error bars represent 1 SD from the mean fold enrichment.

To address whether BexR directly regulates transcription of its target genes, we used chromatin immunoprecipitation (ChIP). We constructed a strain in which the native chromosomal copy of the *bexR* gene has been modified to encode a version of BexR containing a vesicular stomatitis virus glycoprotein (VSV-G) epitope tag at its C-terminus (BexR-V). This strain retained the ability to bistably express PA1202 *lacZ* on LB agar containing X-Gal, suggesting that the VSV-G epitope tag does not interfere with BexR activity (data not shown). We immunoprecipitated BexR-V-associated DNA from wild-type ON cultures grown to both mid-logarithmic and stationary phase and quantified occupancy of BexR-V at candidate target promoters relative to a control region not expected to bind BexR-V. BexR-V strongly occupies its own promoter, as well as those of PA1202 and PA0572 (Figure 3). Furthermore, BexR-V occupied the *aprX* and *aprA* promoters, but not the intervening DNA upstream of *aprD*. This suggests that BexR-V has at least two distinct binding sites within the *apr* locus. All occupancies were significantly higher than those observed in both wild-type OFF cultures and in a non-epitope tagged control strain (Figure S3). These results suggest that BexR regulates many of its target genes directly.

### Positive Feedback of *bexR* Is Required for Bistability

The evidence presented thus far suggests that *bexR* encodes a bistably expressed transcription regulator that positively regulates its own expression. This is reminiscent of the competence switch in *B*. *subtilis*. In this system, ComK, the master regulator of competence, positively regulates transcription of its own gene, thereby enabling a non-linear response to increasing concentrations of ComK, which leads to bistability in the development of competence. Using single-cell fluorescent reporter analysis, it has been shown that the ComK positive feedback loop is required for bistable expression of competence [14],[15]. We hypothesized that, in a similar manner, the positive feedback loop controlling *bexR* expression is required for bistable expression of the BexR regulon (i.e. positive feedback of *bexR* creates a condition of hypersensitivity to variation in levels of BexR protein). If this hypothesis is correct a gradual increase in basal *bexR* expression should increase the proportion of ON relative to OFF cells specifically in a strain with an intact positive feedback loop. In a strain that lacks this positive feedback loop, a graded increase in *bexR* expression should lead to a corresponding increase in expression of *bexR*-regulated genes with no detectable bistability.

**Figure 4:**
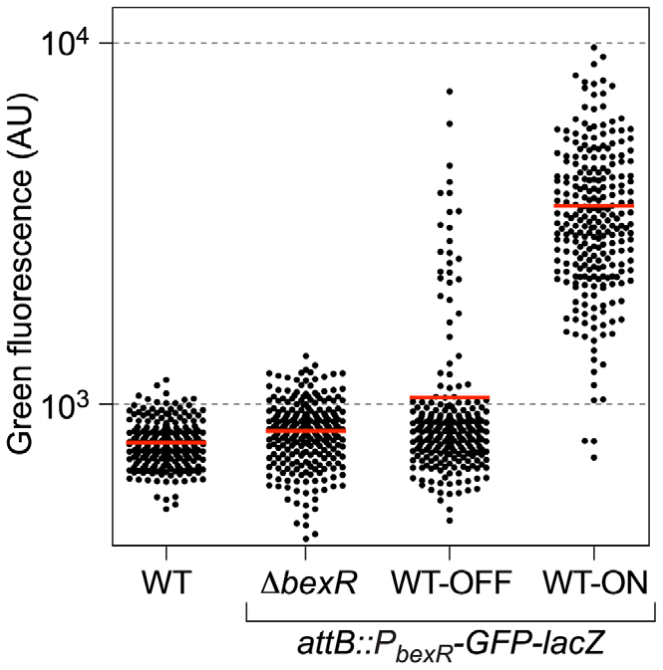
Automated fluorescence intensity measurement of single cells reveals bistable expression from the *bexR* promoter. Cultures of wild-type PAO1 (WT), the wild-type *attB::P_bexR_-GFP-lacZ* fluorescent reporter strain (originating from ON and OFF colonies) and the Δ*bexR attB::P_bexR_-GFP-lacZ* fluorescent reporter strain (see Figure 1D), were grown and examined and measured for fluorescence upon reaching mid-logarithmic phase by fluorescence microscopy. Black dots correspond to the automatically measured fluorescence intensity of individual cells (in arbitrary units, AU) in a sample size of 250 cells. The red bar represents the mean fluorescence intensity of cells in a sample.

**Figure 5:**
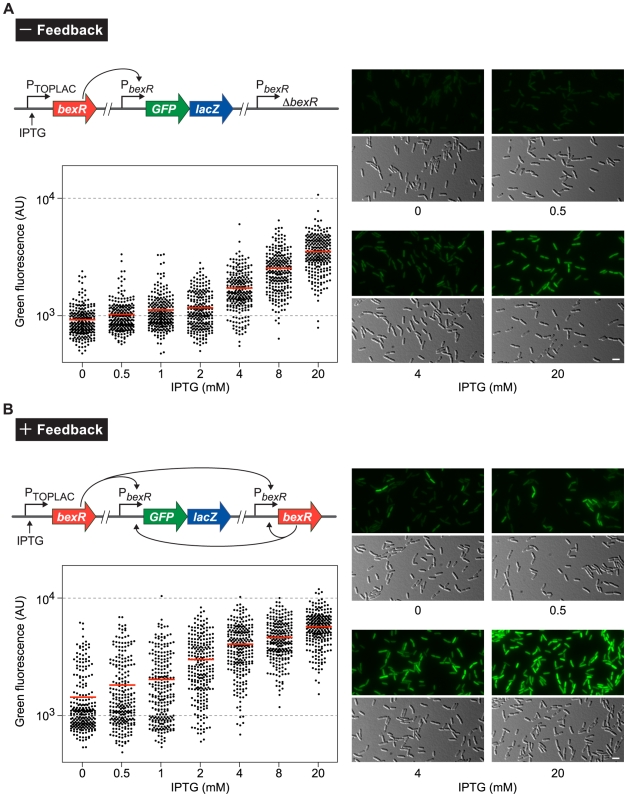
Positive feedback of *bexR* is required for bistability of the regulon. Cells of both the minus feedback strain (PAO1 Δ*bexR attB::P_bexR_-GFP-lacZ attTn7::TOPLAC-bexR*, see diagram in (A), and the plus feedback strain (PAO1 *attB::P_bexR_-GFP-lacZ attTn7::TOPLAC-bexR*, see diagram in (B) were grown in LB media containing IPTG at the indicated concentrations, and examined and measured for fluorescence upon reaching mid-logarithmic phase by fluorescence microscopy. Black dots on scatter plots correspond to the automatically measured fluorescence intensity of individual cells (in arbitrary units, AU) in a sample size of 250 cells. The red bar represents the mean fluorescence intensity of cells in a sample. Representative micrographs of selected samples are shown, with green fluorescence displayed in pseudocolor on the top panels and the corresponding DIC image on the bottom panels. Scale bar, 3 µm.

**Figure 6:**
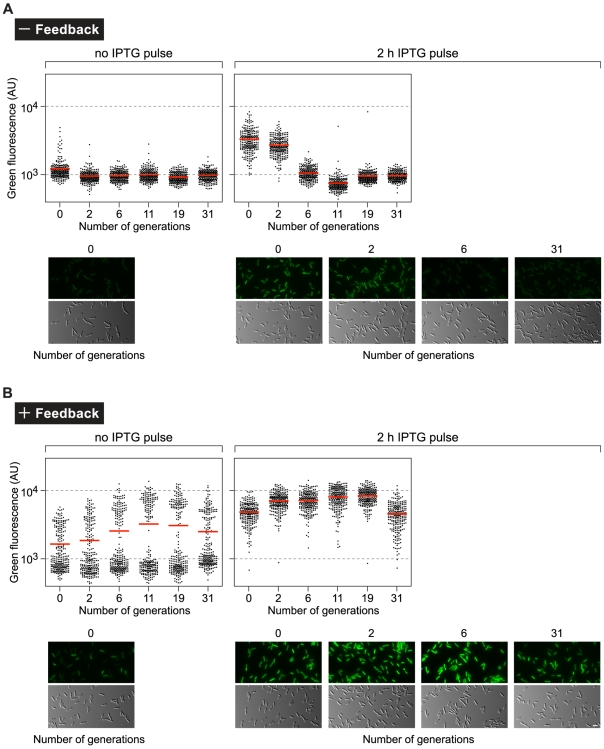
The feedback-mediated BexR switch exhibits hysteresis. Cells of both the minus feedback strain (PAO1 Δ*bexR attB::P_bexR_-GFP-lacZ attTn7::TOPLAC-bexR*, see diagram, Figure 5A), and the plus feedback strain (PAO1 *attB::P_bexR_-GFP-lacZ attTn7::TOPLAC-bexR*, see diagram, Figure 5B) were grown to early logarithmic phase and were exposed to a pulse of IPTG (20 mM) for 2 hours (2 h IPTG pulse) to induce ectopic expression of *bexR*, or not exposed to IPTG (no IPTG pulse). Cells were then washed (to remove IPTG) and grown in fresh media for 31 generations. Cells were examined and measured for fluorescence after 0, 2, 6, 11, 19, and 31 generations post treatment with or without IPTG by fluorescence microscopy. For scatter plots, the number of generations of growth in media without IPTG after the pulse is given on the horizontal axes. Black dots correspond to the automatically measured fluorescence intensity of individual cells (in arbitrary units, AU) in a sample size of 250 cells. The red bar represents the mean fluorescence intensity of cells in a sample. Representative micrographs of selected samples are shown, with green fluorescence displayed in pseudocolor on the top panels and the corresponding DIC image on the bottom panels. Scale bar, 3 µm.

Wild-type *P*. *aeruginosa* cells containing a *P_bexR_-GFP-lacZ* reporter construct integrated in single copy into the chromosome can be seen to exhibit BexR-dependent bistable expression of this reporter by fluorescence microscopy (Figure 1E). Consistent with this observation, quantification of the fluorescence of individual cells within a culture derived from either an ON colony or an OFF colony reveals that cells in the ON and OFF expression states can be distinguished from one another, and that each culture contains both ON and OFF cells (Figure 4). To analyze the effect of positive feedback on *bexR* bistability, we constructed a pair of strains containing the *P_bexR_-GFP-lacZ* reporter construct and an isopropyl-β-D-thiogalactoside (IPTG)-inducible copy of *bexR* (also provided in single copy from the chromosome from a different locus). One of these strains contained an unmarked, in-frame deletion of *bexR* (the minus feedback strain, Figure 5A), whereas the other contained wild-type *bexR* at its native locus (the plus feedback strain, Figure 5B). In the absence of IPTG, only cells of the reporter strain with the intact positive feedback loop displayed bistability, and contained two populations of cells corresponding to those in the ON and OFF expression states (manifest in Figure 5B [and Figure 6B] as a population of cells with an essentially bimodal distribution of fluorescence intensities). Furthermore, a gradual increase in ectopically expressed *bexR* resulted in an increase in the proportion of ON relative to OFF cells only in the plus feedback strain (Figure 5B); in the strain lacking the positive feedback loop, cells responded relatively uniformly to increasing synthesis of ectopically expressed *bexR* (manifest in Figure 5A as a population of cells with a normal distribution of fluorescence intensities, whose average fluorescence intensity increases with IPTG concentration). Importantly, for IPTG concentrations at which the average cell fluorescence intensity was similar between cells with and without feedback, two distinct cell populations (ON and OFF) were observed only in cells with an intact positive feedback loop (Figure 5). In particular, cells of the plus feedback strain at 0.5 mM IPTG had a mean fluorescence intensity of 1814 arbitrary units, which is similar to the mean fluorescence intensity of 1720 arbitrary units exhibited by the minus feedback strain at 4 mM IPTG. Whereas the mean reporter gene expression of these two cell populations, and thus the average abundance of BexR protein per cell, was quite similar under these two conditions, the existence of two subpopulations of cells occurred only in the presence of *bexR* autoregulation (Figure 5). These results suggest that positive feedback of *bexR* is necessary for bistability.

**Figure 7:**
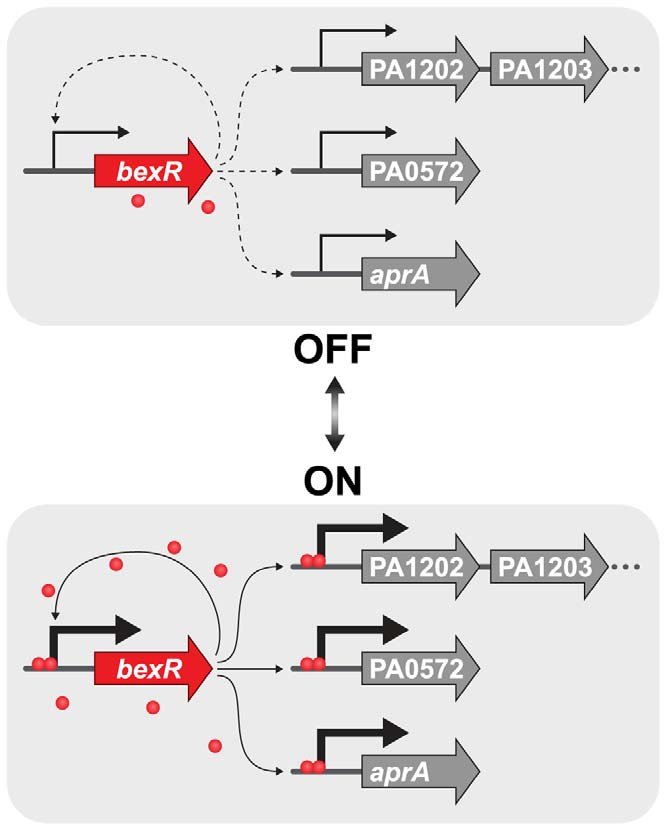
A model for the switch to the ON state. Wild-type *P*. *aeruginosa* is hypersensitized to BexR levels by virtue of positive feedback at the *bexR* locus. Cell-to-cell variability in basal *bexR* expression results in a stochastically determined subset of cells activating the positive autoregulatory loop by BexR binding to its own promoter and activating transcription. At this point, the ON state is maintained by direct positive feedback. Transcription of downstream genes such as *aprA*, PA0572, and the PA1202 operon is upregulated in the ON state by BexR binding to their promoters and activating transcription.

Feedback-mediated bistable systems often exhibit a capacity for history-dependent behavior, or hysteresis [reviewed in 23]. Systems exhibiting hysteretic behavior may have different responses under identical conditions, depending on the conditions previously experienced. For example, in bistable expression of the *lac* operon of *E*. *coli* at low concentrations of a non-metabolizable lactose analog, the concentration of inducer at which initially uninduced cells turn on is higher than that at which initially induced cells turn off [24],[25]. The behavior of this system at concentrations of inducer between these thresholds therefore depends on conditions previously encountered. Thus, systems with positive feedback can exhibit memory of previous expression states. To investigate the possibility that positive feedback of *bexR* can impart a memory of previous expression states on the system, we utilized the plus and minus feedback strains described above (Figure 5) and observed their response over time to a pulse of ectopically expressed *bexR*, induced by a 2 hour exposure to 20 mM IPTG. In cells without an intact positive feedback loop, the IPTG pulse was sufficient to raise the mean fluorescence intensity to the level seen in wild-type ON cells (Figure 6A and Figure 4). However, this degree of expression from the *P_bexR_-GFP-lacZ* reporter was quickly lost upon removal of IPTG and subculturing of cells into fresh media. In contrast, cells of the plus feedback strain maintained their induced state for many generations after the removal of IPTG, suggesting that a brief period in which cells experience a high intracellular concentration of BexR is sufficient to induce a long-lasting ON state (Figure 6B). Indeed, a pulse with IPTG for only 30 minutes is sufficient to induce a transition to a sustained ON state in the plus feedback strain (Figure S4). Only after 31 generations following removal of IPTG, do a portion of the cells begin to transition to the OFF state (Figure 6B). Taken together, the results of our single-cell population analyses suggest a mechanism in which variation in basal expression of *bexR* in OFF cells is amplified by a positive feedback loop in a stochastically determined subset of cells that then transitions to the ON state and is maintained in that state by continued autoactivation of BexR (Figure 7).

## Discussion

The results above characterize a heretofore undescribed bistable switch in *P*. *aeruginosa* that controls virulence gene expression. We have shown that *bexR*, which encodes a LysR-type transcription regulator, is bistably expressed, and that this bistability results in altered expression of several downstream genes, including those in the uncharacterized PA1202 operon and *aprA*, which encodes the virulence factor alkaline protease. Furthermore, reporter assays show that BexR can positively regulate its own expression. ChIP analysis indicates that BexR acts directly at the sites of many target promoters, including that of its own gene. Finally, single-cell analyses of the response of a cell population to a graded source of BexR, or a pulse of BexR, suggests that positive autoregulation is necessary for the observed bistability. Taken together, these results outline a novel feedback-mediated bistable switch in an opportunistic pathogen.

### Phenotypic Outcomes of BexR Bistability

Bistability is a mechanism by which bacteria can introduce phenotypic heterogeneity within an isogenic population, thereby creating a subset of cells capable of surviving the onset of an otherwise lethal situation. For example, some bacteria have the ability to survive antibiotic treatment without evolving *bona fide* resistance by stochastically entering a dormant “persister” state during vegetative growth [26]. A recent study suggests that a *bexR* transposon mutant has 2-fold increased sensitivity to the fluoroquinolone antibiotic ciprofloxacin, which is used in treatment of *P*. *aeruginosa* infections in CF patients, though the potential mechanism for this increased sensitivity was not addressed [27],[28]. Although our findings raised the possibility that bistable *bexR* expression might lead to heterogeneity in ciprofloxacin resistance, we found no evidence that *bexR* contributed to the resistance of *P. aeruginosa* to ciprofloxacin, at least in strain PAO1 (data not shown).

Bistable expression of virulence factors has been previously reported in *P*. *aeruginosa*. For instance, the Type III secretion system is only expressed in a subset of cells grown in inducing conditions [29]. Additionally, the *cupA* fimbrial gene cluster is bistably expressed by *P*. *aeruginosa* when grown in anaerobic conditions [5]. Bistable expression of several virulence factors independently of one another may create several subtypes of cells with differing virulence potential within an isogenic population of infecting bacteria. Thus, bistable expression of virulence factors may represent a strategy employed by *P*. *aeruginosa* to generate cell types specialized to survive within different niches in the host.

In *P*. *entomophila*, AprA has a significant role in virulence in a *D*. *melanogaster* oral model of infection, where it is thought to protect the bacterium from the effects of host-produced antimicrobial peptides [22]. Although oral models of *D*. *melanogaster* infection with *P*. *aeruginosa* have been used to successfully characterize bacterial virulence, these models have not been used to test the role of AprA in *P*. *aeruginosa* virulence [30],[31]. If alkaline protease does play a role in defense against antimicrobial peptides in *P*. *aeruginosa*, upregulating *aprA* ∼2-fold in a subset of cells through BexR-mediated bistability may preemptively adapt a portion of the cell population to the sudden introduction to a particular host environment. *P*. *aeruginosa* alkaline protease has been shown to degrade a variety of human proteins and tissues and inhibit immune cell function, presumably by acting at the cell surface to modify phagocytic and chemotactic receptors (reviewed in [32]). Alkaline protease has also been suggested to play a role in corneal keratitis [33], although this role for AprA has been disputed more recently by the comparison of isogenic mutant strains [34]. However, our observation that wild-type strains of *P*. *aeruginosa* bistably express *aprA* may complicate the interpretation of earlier work. Interestingly, the rhizobacterium *P*. *brassicacearum* exhibits phenotypic variation in expression of an alkaline protease homolog, though whether this is mediated by bistability of a BexR homolog is unknown [35]. It has been suggested that heterogeneous production of extracellular proteases by an isogenic population of bacteria is an example of cooperative or altruistic behavior, as these proteases diffuse freely through the growth medium and can equally benefit all members of the population [36]. Thus, bistable production of alkaline protease or PA0572, a predicted protease, may serve to benefit both ON and OFF cells in a population. Whether bistable expression of *aprA*, or other members of the BexR regulon, has a role in mammalian virulence remains to be seen.

In contrast with *aprA*, many other regulatory targets of BexR are poorly characterized hypothetical genes. BexR-mediated bistability does not appear to be limited to *P*. *aeruginosa* PAO1, as the homolog of PA1202 in *P*. *aeruginosa* PA14, a more virulent clinical strain, is also bistably expressed in a BexR-dependent manner (Figure S5). This conservation across diverse strains of *P*. *aeruginosa* suggests an important biological role for BexR-mediated bistability. In this regard, a particularly interesting target of BexR is the PA1202 operon, which is strongly positively regulated by BexR. Several genes in this operon, such as PA1202 and PA1205, are predicted to encode enzymes with catabolic activity directed against small molecules. This may point to a role for the BexR regulon in the ability of *P. aeruginosa* to metabolize and thereby detoxify certain small molecules. Co-regulation of a diverse set of genes by BexR may indicate that it is involved in manifestation of more than one phenotype. That these genes are expressed in a bistable manner suggests that their expression or repression may be detrimental to growth under certain conditions.

### Feedback-Mediated Bistability and the BexR Switch

We propose that positive feedback of *bexR* provides a mechanism for amplification and propagation of cell-to-cell variability in BexR levels. This regulatory circuit is similar to the one governing competence development in *B*. *subtilis*. Experiments in this system have suggested that noisy expression of *comK* results in ComK levels in a subpopulation of cells crossing a threshold level for *comK* autoactivation, causing differentiation into the competent state [14],[15],[37]. Noise in *bexR* expression may also provide the basis for generating cell-to-cell variability in BexR levels. The frequency of the BexR switch differs from that of the ComK switch. Whereas *B*. *subtilis* has been directly observed to enter a competent state in approximately 3.6% of cell division events [38], *P*. *aeruginosa* enters into the BexR-ON state approximately 10-fold less frequently, and the BexR-OFF state even less so (Table 1 and Table S1). These low frequencies are on par with classical phase-variation systems, but in the case of BexR, the expression state stability appears to be epigenetically mediated. This low switching frequency may be a function of the high degree of hysteresis observed in the BexR switch. Biological systems capable of hysteretic behavior can retain a memory of previous exposure to inducing conditions, and this has been observed in both naturally occurring and synthetic systems [25],[39]. Strictly speaking, hysteresis is not a necessary characteristic of bistable systems, as a synthetic feedback-mediated bistable system was observed to exhibit clear bistability but display no history-dependent response [40]. Nevertheless, hysteresis is often associated with bistable systems, and that it is observed in the BexR switch may suggest that retaining memory of previous conditions is beneficial to the cell.

In *B*. *subtilis*, regulation of ComK levels is achieved by degradation of ComK by the MecA/ClpCP complex and the inhibition thereof by ComS [41],[42]. Our single-cell population analyses indicate that directly modulating BexR levels by induction of ectopic synthesis can affect the frequency at which cells differentiate into the ON state (Figure 5). Modulation of BexR levels or activity in wild-type cells may provide a mechanism for fine-tuning the dynamics of this bistability. There may be accessory factors, perhaps themselves BexR-regulated, that affect BexR levels or activity. A mechanism for modulating BexR autoactivation dynamics may allow *P*. *aeruginosa* to regulate switching frequency in response to external conditions. As LysR-type transcription activators often bind to small molecules to alter their DNA-binding and regulatory properties, it is possible that the dynamics of the BexR switch may be tunable by a coinducer molecule [43]. However, no such molecule has yet been identified.

The results presented here outline a model for differentiation into the BexR-ON state, but do not address the mechanism by which a BexR-ON cell can revert to the BexR-OFF state. Previous studies suggest that escape from a positive feedback loop is often mediated by an accessory process. For example, escape from competence in *B*. *subtilis* occurs when reduction in ComS levels promotes ComK proteolysis by the MecA/ClpCP complex, relieving ComK autoactivation [38]. The switch from BexR-ON to BexR-OFF may also involve some antagonistic process. Unlike several other feedback-mediated bistable switches, the switch from ON to OFF in the case of BexR appears to occur only in a stochastically determined subset of cells. For example, escape from competence in *B*. *subtilis* occurs because *comS* transcription is repressed by ComK in the competent state and ComS protein gradually depletes in all cells [38]. In contrast, the BexR-ON state is relatively stable and heritable, and is lost only in a subpopulation of cells. The existence of a stochastic process mediating the switch to BexR-OFF that is distinct from the one mediating the switch to BexR-ON, is further supported by the ∼60-fold directional bias in switching frequencies (Table 1). This process may take the form of transcription regulation of *bexR* or post-translational modulation of BexR levels or activity, and we are currently investigating these possibilities.

## Materials and Methods

### Bacterial Strains and Plasmids

*P. aeruginosa* strains PAO1 and PA14 were provided by Arne Rietsch (Case Western Reserve University). *E. coli* DH5α F'IQ (Invitrogen) was used as the recipient strain for all plasmid constructions, whereas *E. coli* strain SM10 (λpir) was used to mate plasmids into *P. aeruginosa*.

The PA1202 *lacZ* reporter strain (PAO1 *PA1202 lacZ*) contains the *lacZ* gene integrated immediately downstream of the PA1202 gene on the PAO1 chromosome and was made by allelic exchange. PCR products 486 bp and 513 bp in length flanking the 3′ end of PA1202 were amplified and spliced together to add *Kpn*I, *Nco*I and *Sph*I sites two bases after the PA1202 stop codon. This PCR product was cloned as a *Sac*I/*Pac*I fragment into pEXG2 [44]. The *lacZ* gene was subsequently cloned into this construct as a *Kpn*I/*Sph*I fragment derived from pP18-*lacZ* (Arne Rietsch, unpublished work), generating plasmid pEXF1202-*lacZ*. This plasmid was then used to create reporter strains PAO1 *PA1202 lacZ* and PA14 *PA1202 lacZ* by allelic exchange.

The deletion construct for the *bexR* gene (PA2432) was generated by amplifying regions 398 bp and 360 bp in length that flank *bexR* in the PAO1 genome by the PCR and then splicing the flanking regions together by overlap extension PCR; deletions were in-frame and contained the 9-bp linker sequence 5′-GCGGCCGCC-3′. The resulting PCR product was cloned on an *Eco*RI/*Bam*HI fragment into plasmid pEX18Gm [45], yielding plasmid pEXM2432. This plasmid was then used to create strains PAO1 Δ*bexR*, PAO1 *PA1202 lacZ* Δ*bexR* and PA14 *PA1202 lacZ* Δ*bexR* by allelic exchange [45]. Deletions were confirmed by the PCR.

The *attB::P_bexR_-lacZ* reporter strains contain fragments of the *bexR* promoter fused to the *lacZ* gene and integrated in single copy into the *attB* locus in the PAO1 chromosome and were made by site-specific integration followed by backbone excision through transient synthesis of FLP recombinase from plasmid pFLP2 [17],[45]. PCR products spanning from 91, 198 or 297 bp to 3 bp upstream of the *bexR* start codon were amplified and cloned as *Eco*RI/*Xho*I fragments into mini-CTX-*lacZ* [17], which contains a consensus Shine-Dalgarno sequence upstream of *lacZ*, yielding plasmids mini-CTX-*PF2432*-*lacZ*.1, mini-CTX-*PF2432*-*lacZ*.2 and mini-CTX-*PF2432*-*lacZ*.3, respectively. These plasmids were then used to create reporter strains PAO1 *attB::P_bexR_*.1-*lacZ*, PAO1 Δ*bexR attB::P_bexR_*.1-*lacZ*, PAO1 *attB::P_bexR_*.2-*lacZ*, PAO1 Δ*bexR attB::P_bexR_*.2-*lacZ*, PAO1 *attB::P_bexR_*.3-*lacZ* and PAO1 Δ*bexR attB::P_bexR_*.3-*lacZ*. An *Eco*RI/*Xho*I fragment of mini-CTX-*PF2432*-*lacZ*.3 was subcloned into mini-CTX-GFP-*lacZ* [16], yielding plasmid mini-CTX-*PF2432*-GFP-*lacZ*.3. This plasmid was then used to create the fluorescent reporter strains PAO1 *attB::P_bexR_*.3-GFP-*lacZ* and PAO1 Δ*bexR attB::P_bexR_*.3-GFP-*lacZ*.

The BexR-VSV-G integration vector was generated by first cloning a PCR-amplified DNA fragment containing ∼300 bp of sequence from the 3′ portion of the *bexR* gene on a *Hin*dIII/*Not*I fragment into plasmid pP30Δ-YTAP [4], generating plasmid pP30Δ-BexR-TAP. This *Hin*dIII/*Not*I fragment was then subcloned into pP30ΔFRT-MvaT-V [46], generating plasmid pP30ΔFRT-BexR-V. This plasmid was used to make strain PAO1 *PA1202 lacZ* BexR-V by homologous recombination at the *bexR* locus followed by backbone excision through transient synthesis of FLP recombinase from plasmid pFLP2 [45]. Production of the BexR-V protein was confirmed by Western blotting with an anti-VSV-G antibody (Sigma).

Plasmid pBexR is a derivative of pPSV35 [44] and directs the synthesis of the BexR protein under control of the IPTG-inducible *lacUV5* promoter. The plasmid was made by subcloning an *Eco*RI/*Hin*dIII DNA fragment containing a consensus Shine-Dalgarno sequence and the *bexR* gene into pPSV35.

The *attTn7::TOPLAC*-*bexR* strains contain a construct which directs the synthesis of the BexR protein under control of the IPTG-inducible *TOPLAC* promoter stably integrated into the genome in single copy at the *attTn7* locus. The *TOPLAC* promoter in this construct is a derivative of the *lac* promoter that contains two *lac* operator sequences centered at positions −63.5 and +11. The sequence of this promoter is 5′-CACTACGTGCTCGAGGGT**AAATGTGAGCACTCACAATT**TATTCTGAAATGAGCTCTTTACACGTCCTGCTGCCGGCTCGTATGTTGTGTGG**AATTGTGAGCGGATAACAATT**AAGCTTAGTCGACAGCTAGCCGGATCC-3′, where the -35 and -10 sequences are underlined and the *lac* operator sequences are shown in bold. The *bexR* gene is inserted downstream of the *TOPLAC* promoter with a consensus Shine-Dalgarno sequence. This construct was inserted between the ends of the Tn7 transposon on pUC18-mini-Tn7T-LAC [47], generating plasmid pUC18-mini-Tn7T-TOPLAC-*bexR*. This plasmid was used to make strains PAO1 *attB::P_bexR_*.3-GFP-*lacZ attTn7::TOPLAC*-*bexR* and PAO1 Δ*bexR attB::P_bexR_*.3-GFP-*lacZ attTn7::TOPLAC*-*bexR* by site-specific recombination [47].

### β-Galactosidase Assays

Cells were grown with aeration at 37°C to mid-logarithmic phase in LB supplemented as needed with gentamicin (25 µg/ml) and IPTG (0.1 mM). Cells were permeabilized with sodium dodecyl sulfate and CHCl_3_ and assayed for *β*-galactosidase activity as described previously [48]. Assays were performed at least twice in triplicate on separate occasions. Representative data sets are shown.

### RNA Isolation

Cultures of PAO1 Δ*bexR attB::P_bexR_.3-lacZ* and PAO1 *attB::P_bexR_.3-lacZ* in the OFF and ON states were inoculated in quadruplicate at starting OD_600_ of ≈0.01 and grown with aeration to an OD_600_ of ≈0.55 (representing mid-logarithmic phase) and to an OD_600_ of ≈2.4 (representing stationary phase) at 37°C in LB. Cells were then harvested by centrifugation and RNA prepared essentially as described [49]. Transcripts were quantified by quantitative real-time RT-PCR as described [50].

### Switching-Frequency Calculations

Switching-frequency calculations were performed essentially as described [51], except that cells were plated on LB agar plates containing 50 µg/ml X-Gal and grown at 37°C. Error values represent 1 standard deviation (SD) from the mean switching frequency.

### Microarray Experiments

Cultures of PAO1 Δ*bexR* containing plasmid pPSV35 [44] or pBexR were grown with aeration at 37°C in LB containing gentamicin (25 µg/ml). Triplicate cultures of each strain were inoculated at a starting OD_600_ of ≈0.01 and grown to an OD_600_ of ≈0.5 (representing mid-logarithmic phase) and to an OD_600_ of ≈2.3 (representing stationary phase). RNA isolation, cDNA synthesis, and cDNA fragmentation and labeling were performed essentially as described previously [49]. Labeled samples were hybridized to Affymetrix GeneChip *P*. *aeruginosa* genome arrays (Affymetrix). Data were analyzed for statistically significant (p<0.05, fold change >2) changes in gene expression using GeneSpring GX.

### Chromatin Immunoprecipitation (ChIP)

Cultures of PAO1 *PA1202 lacZ* BexR-V in either the ON or OFF state were inoculated in quadruplicate at a starting OD_600_ of ≈0.01 and grown with aeration to an OD_600_ of ≈0.5 (representing mid-logarithmic phase) and to an OD_600_ of ≈2.0 (representing stationary phase) at 37°C in LB. ChIP was then performed with 3 ml of culture and fold enrichment values were measured by quantitative PCR relative to the PA2155 promoter essentially as described [46].

### Quantitative Fluorescence Microscopy

For fluorescence micrograph analysis, cultures were fixed with formaldehyde and glutaraldehyde at 2.4% and 0.04%, respectively, and cells were allowed to fix for 30 minutes at room temperature. Cells were washed three times with PBS and imaged on a Nikon TE2000 inverted microscope outfitted with a Nikon Intensilight illuminator, a Coolsnap HQ2 charge-coupled device camera from Photometrics and a Nikon CFI Plan Apo VC ×100 objective lens (1.4 NA) for differential interference contrast (DIC) imaging. For GFP images the ET-GFP filter set (Chroma 49002) was used. Images were captured using Nikon Elements software, which was also used for quantification of fluorescence in individual cells. This was done by automatically defining cell boundaries using the DIC image, excluding cells that were poorly focused, narrower than 0.5 µm, longer than 4.0 µm or shorter than 0.5 µm, and using those regions to quantify the GFP image. Values given are subtracted for background fluorescence. At least 400 cells were imaged for each timepoint, and the fluorescence intensities of a random subset of 250 cells are displayed in scatter plots. Images were exported to Adobe Photoshop CS4 for preparation.

For the hypersensitivity experiment (Figure 5), cells were grown with aeration at 37°C to mid-logarithmic phase in LB supplemented as needed with IPTG and prepared for microscopy as described above. The experiment was performed at least twice in duplicate on separate occasions. A representative data set from a single replicate is shown.

The hysteresis experiment (Figure 6) was performed by growing cells with aeration at 37°C in LB and either treating them with 20 mM IPTG for 2 hours or 30 minutes immediately before reaching mid-logarithmic phase, or not treating them with IPTG. A sample was then taken and prepared for microscopy (corresponding to the 0 generation time point) as described above while the remaining cells were washed with LB to remove the IPTG, and inoculated into fresh media at a 1∶4 dilution. Cells were then grown continuously for 2 generations to mid-logarithmic phase, a sample was taken and prepared for microscopy (corresponding to the 2 generation time point) and a fresh culture was inoculated at a 1∶16 dilution with the remaining cells. Cells were then grown continuously for 4 generations to mid-logarithmic phase, a sample was taken and prepared for microscopy (corresponding to the 6 generation time point) and a fresh culture was inoculated at a 1∶16 dilution with the remaining cells. Cells were then grown continuously for 5 generations to late-logarithmic phase, a sample was taken and prepared for microscopy (corresponding to the 11 generation time point) and a fresh culture was inoculated at a 1∶32 dilution with the remaining cells. Cells were then grown continuously for 5 generations to late-logarithmic phase and a fresh culture was inoculated at a 1∶16 dilution. Cells were then grown continuously for 3 generations to mid-logarithmic phase, a sample was taken and prepared for microscopy (corresponding to the 19 generation time point), remaining cells were allowed to grow for 1.5 generations to early stationary phase and used to inoculate a fresh culture at a 1∶100 dilution. Cells were then grown continuously for 7 generations (overnight) and used to inoculate a fresh culture at a 1∶100 dilution. Cells were then grown continuously for 3.5 generations to mid-logarithmic phase and a sample was taken and prepared for microscopy (corresponding to the 31 generation time point). The experiment was performed at least three times in duplicate on separate occasions. A representative data set from a single replicate is shown.

## Supporting Information

Figure S1 *bexR* is bistably expressed on solid media in a *bexR*-dependent manner. The *bexR* promoter was fused to *lacZ* and stably integrated in single copy into the PAO1 chromosome. The phenotypes of wild-type and Δ*bexR* strains of this reporter plated on LB agar containing X-Gal are shown.
(1.71 MB TIF)

Figure S2 BexR regulates expression of a diverse set of genes. Cells of PAO1 Δ*bexR* with either empty vector or *bexR*-overexpression vector were grown to mid-logarithmic (ML) and stationary (ST) phase, and mRNA content was profiled by microarray.
(0.09 MB PDF)

Figure S3 BexR occupies the promoters of target genes. Cells of PAO1 *PA1202 lacZ* BexR-V in both ON and OFF states were grown to mid-logarithmic and stationary phase, and DNA associated with BexR-V was analyzed by ChIP. A mock IP control was performed with cells of ON-state PAO1 *PA1202 lacZ* which do not synthesize VSV-G-tagged BexR. Error bars represent one standard deviation from the mean fold enrichment.
(8.17 MB TIF)

Figure S4 A 30 minute pulse of IPTG is sufficient to induce hysteresis. Cells of the plus feedback strain (PAO1 *attB::P_bexR_-GFP-lacZ attTn7::TOPLAC-bexR*, see diagram, Figure 5B) were grown to early logarithmic phase and were exposed to a pulse of IPTG (20 mM) for 30 minutes (30 min IPTG pulse) to induce ectopic expression of *bexR*, or not exposed to IPTG (see Figure 6B, no IPTG pulse). Cells were then washed (to remove IPTG) and grown in fresh media for 31 generations. Cells were examined and measured for fluorescence after 0, 2, 6, 11, 19, and 31 generations post treatment with or without IPTG by fluorescence microscopy. For scatter plots, the number of generations of growth in media without IPTG after the pulse is given on the horizontal axes. Black dots correspond to the automatically measured fluorescence intensity of individual cells (in arbitrary units, AU) in a sample size of 250 cells. The red bar represents the mean fluorescence intensity of cells in a sample. Representative micrographs of selected samples are shown, with green fluorescence displayed in pseudocolor on the top panels and the corresponding DIC image on the bottom panels. Scale bar, 3 µm.
(6.44 MB TIF)

Figure S5 The PA1202-orthologous PA14_48760 operon also exhibits *bexR*-dependent bistability in *P. aeruginosa* strain PA14. (A) Schematic of *PA14_48760 lacZ* reporter strains. (B) Phenotypes of wild-type and Δ*bexR PA14_48760 lacZ* reporter strains when plated on M63 minimal agar containing X-Gal. (C) Quantification of *PA14_48760 lacZ* expression in cultures of the wild-type and Δ*bexR* reporter strains. Error bars represent one standard deviation from the mean β-galactosidase activity.
(3.13 MB TIF)

Table S1 Switching frequencies.
(0.03 MB DOC)
