# Supplementary figures and images for: Epigenetic Control of Virulence Gene Expression in Pseudomonas aeruginosa by a LysR-Type Transcription Regulator
Source: PLoS Genet. 2009 Dec 18;5(12):e1000779. doi: 10.1371/journal.pgen.1000779 (PMC2796861; doi:10.1371/journal.pgen.1000779)

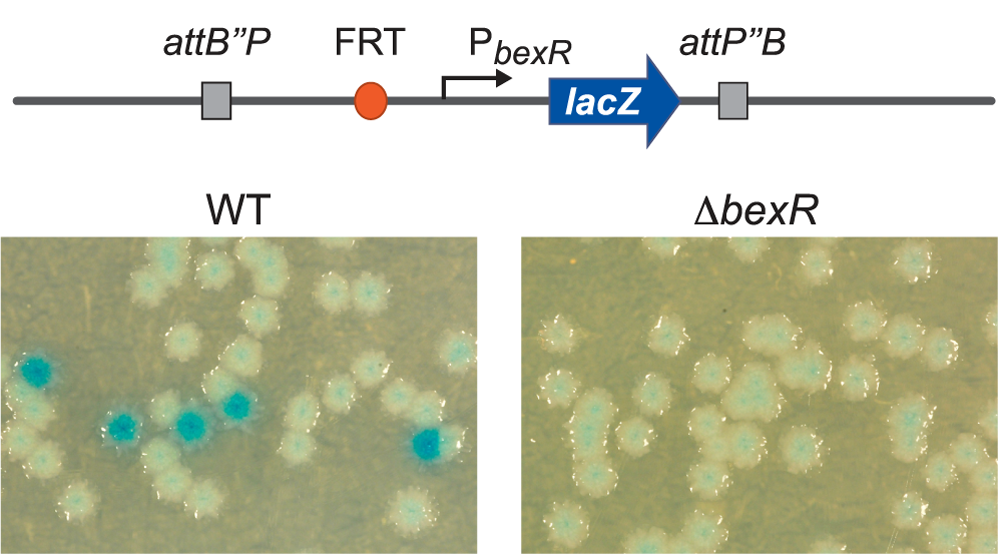

Supplement: Figure S1 — bexR is bistably expressed on solid media in a bexR-dependent manner. The bexR promoter was fused to lacZ and stably integrated in single copy into the PAO1 chromosome. The phenotypes of wild-type and ΔbexR strains of this reporter plated on LB agar containing X-Gal are shown. (1.71 MB TIF) [file pgen.1000779.s001.tif]

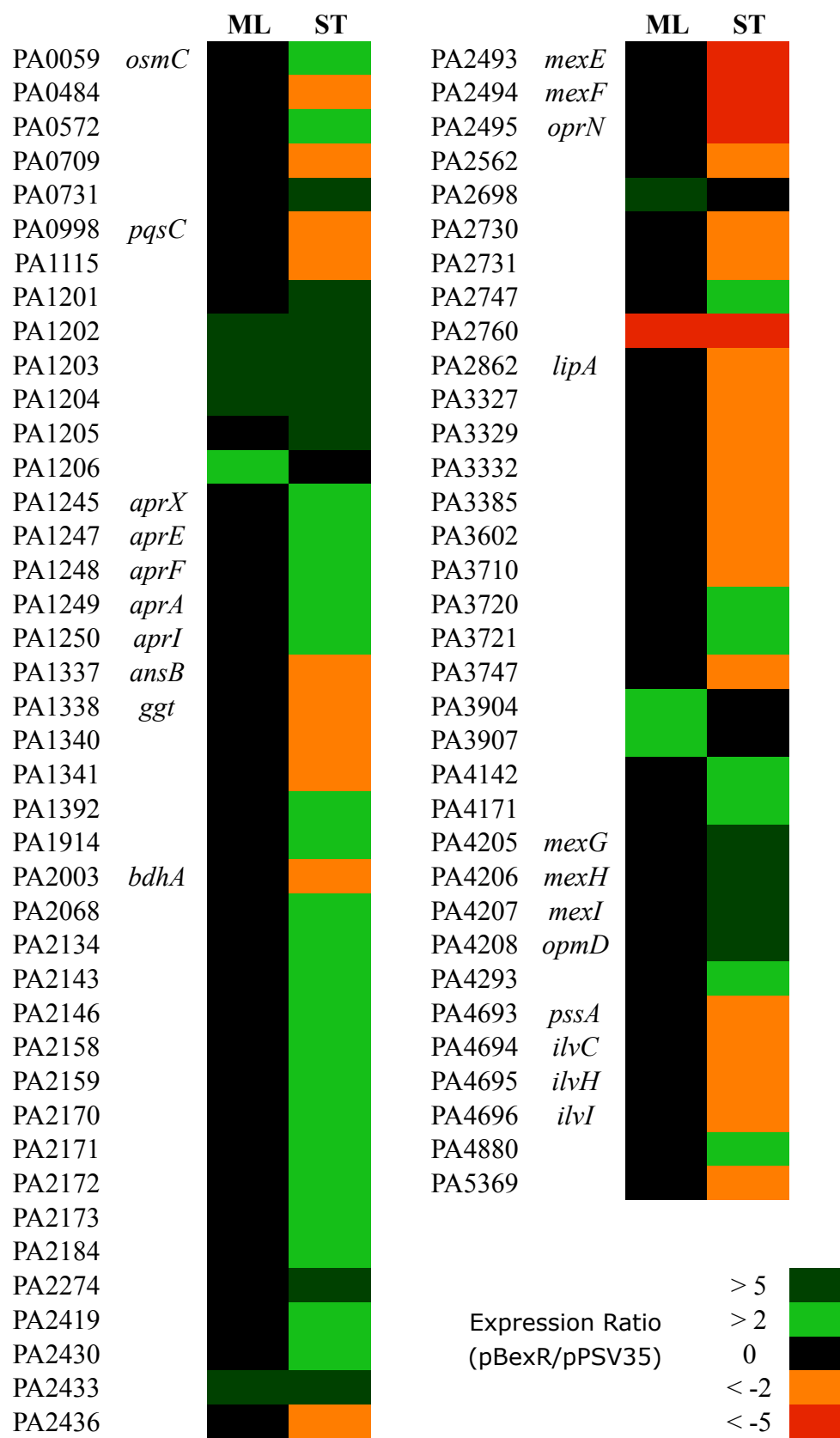

Supplement: Figure S2 — BexR regulates expression of a diverse set of genes. Cells of PAO1 ΔbexR with either empty vector or bexR-overexpression vector were grown to mid-logarithmic (ML) and stationary (ST) phase, and mRNA content was profiled by microarray. (0.09 MB PDF) [file pgen.1000779.s002.pdf]

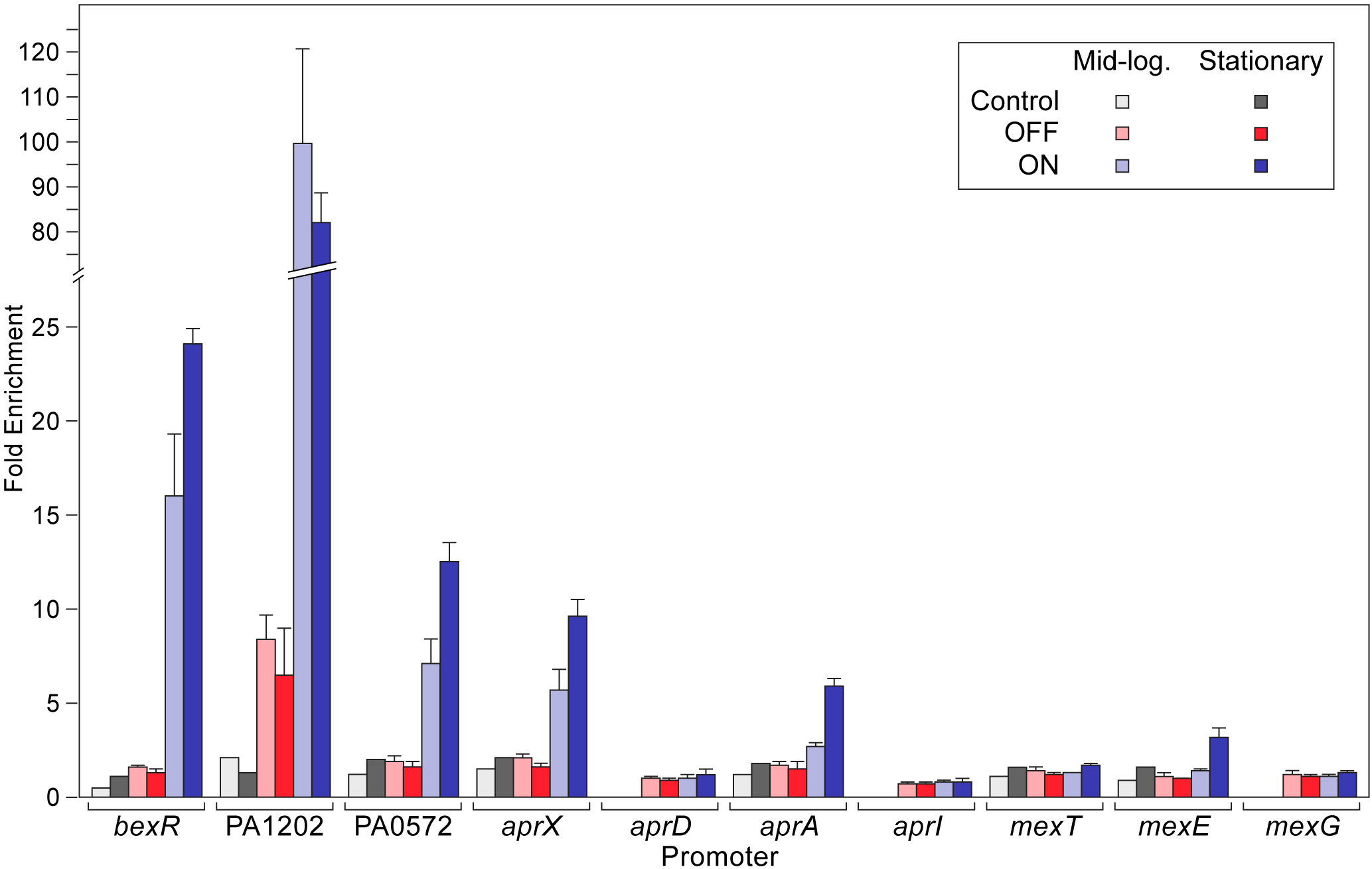

Supplement: Figure S3 — BexR occupies the promoters of target genes. Cells of PAO1 PA1202 lacZ BexR-V in both ON and OFF states were grown to mid-logarithmic and stationary phase, and DNA associated with BexR-V was analyzed by ChIP. A mock IP control was performed with cells of ON-state PAO1 PA1202 lacZ which do not synthesize VSV-G-tagged BexR. Error bars represent one standard deviation from the mean fold enrichment. (8.17 MB TIF) [file pgen.1000779.s003.tif]

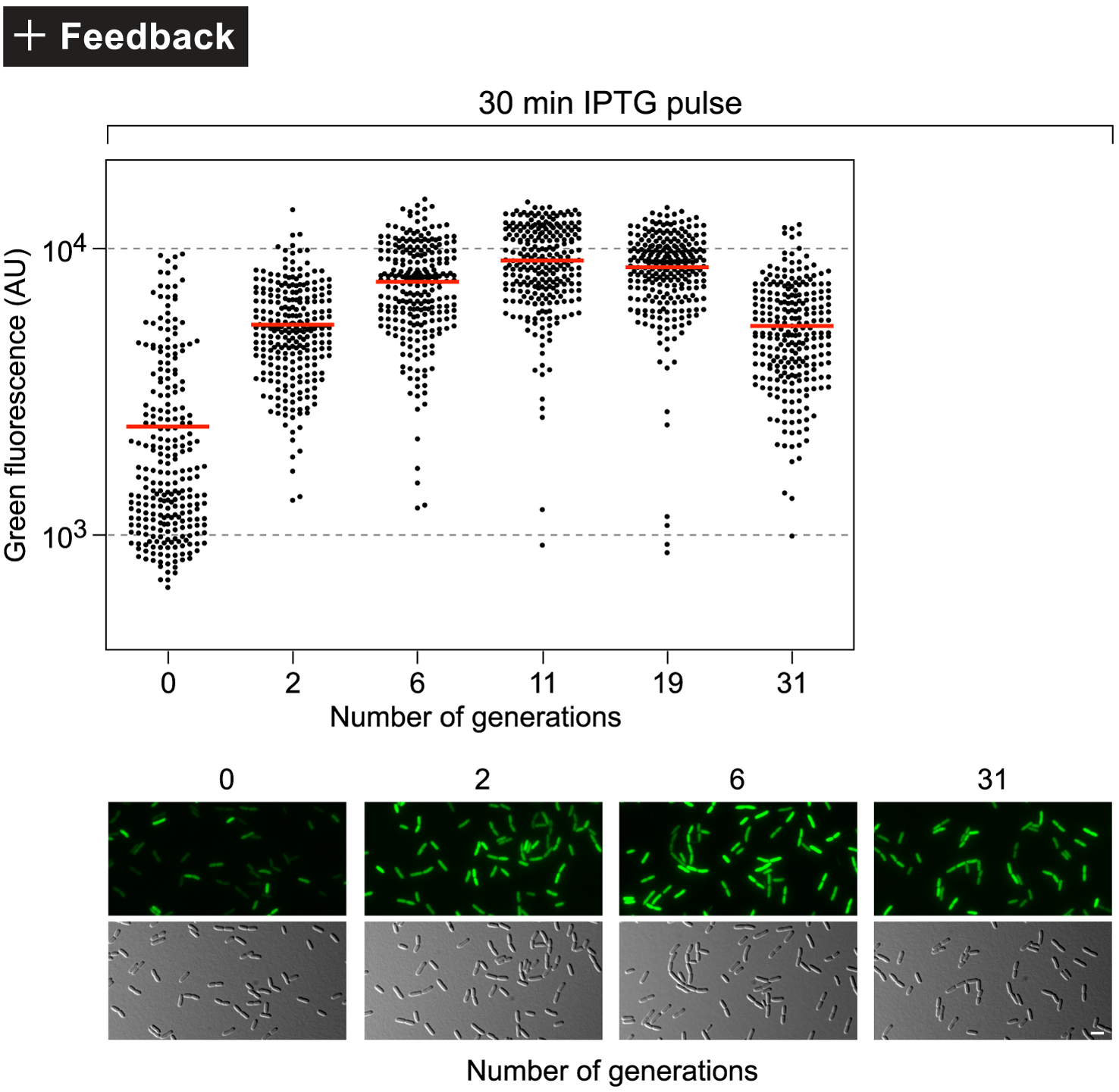

Supplement: Figure S4 — A 30 minute pulse of IPTG is sufficient to induce hysteresis. Cells of the plus feedback strain (PAO1 attB::PbexR-GFP-lacZ attTn7::TOPLAC-bexR, see diagram, Figure 5B) were grown to early logarithmic phase and were exposed to a pulse of IPTG (20 mM) for 30 minutes (30 min IPTG pulse) to induce ectopic expression of bexR, or not exposed to IPTG (see Figure 6B, no IPTG pulse). Cells were then washed (to remove IPTG) and grown in fresh media for 31 generations. Cells were examined and measured for fluorescence after 0, 2, 6, 11, 19, and 31 generations post treatment with or without IPTG by fluorescence microscopy. For scatter plots, the number of generations of growth in media without IPTG after the pulse is given on the horizontal axes. Black dots correspond to the automatically measured fluorescence intensity of individual cells (in arbitrary units, AU) in a sample size of 250 cells. The red bar represents the mean fluorescence intensity of cells in a sample. Representative micrographs of selected samples are shown, with green fluorescence displayed in pseudocolor on the top panels and the corresponding DIC image on the bottom panels. Scale bar, 3 µm. (6.44 MB TIF) [file pgen.1000779.s004.tif]

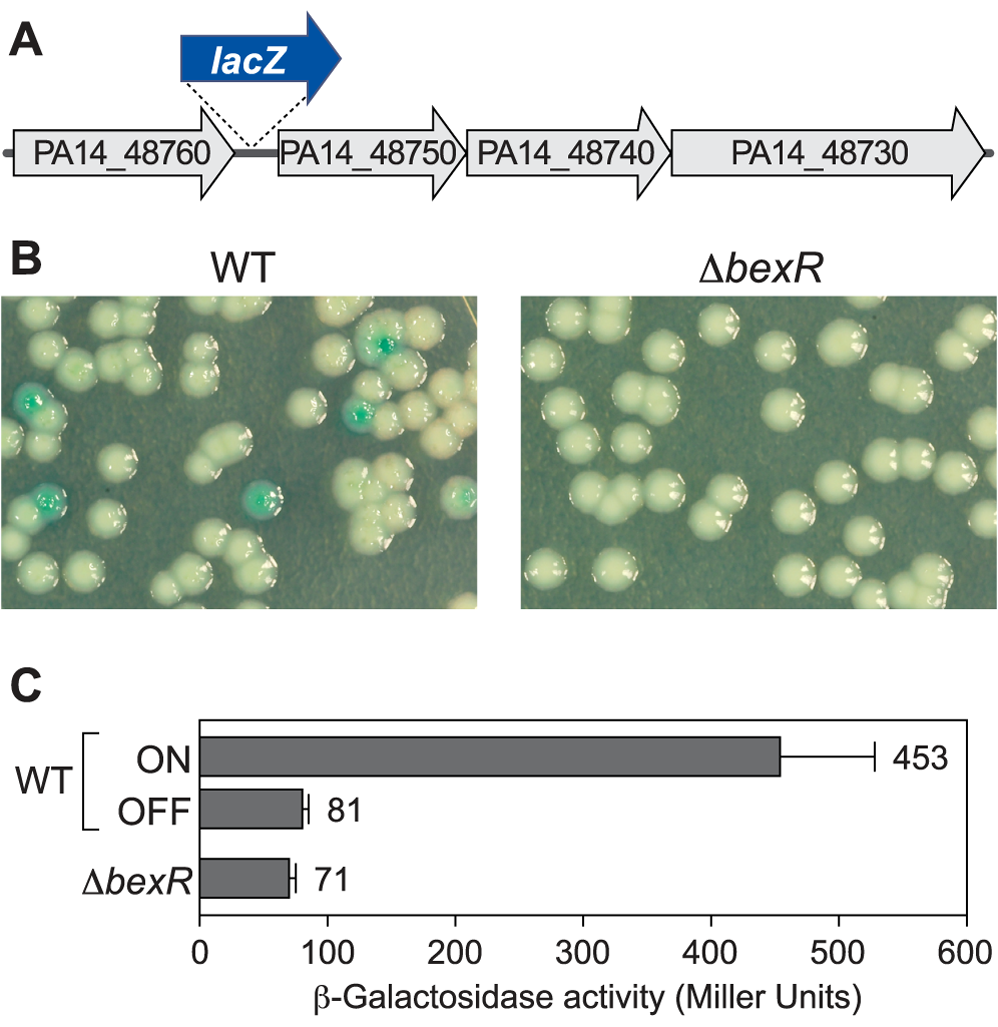

Supplement: Figure S5 — The PA1202-orthologous PA14_48760 operon also exhibits bexR-dependent bistability in P. aeruginosa strain PA14. (A) Schematic of PA14_48760 lacZ reporter strains. (B) Phenotypes of wild-type and ΔbexR PA14_48760 lacZ reporter strains when plated on M63 minimal agar containing X-Gal. (C) Quantification of PA14_48760 lacZ expression in cultures of the wild-type and ΔbexR reporter strains. Error bars represent one standard deviation from the mean β-galactosidase activity. (3.13 MB TIF) [file pgen.1000779.s005.tif]
