# Supplementary material for: Epigenetic Control of Virulence Gene Expression in Pseudomonas aeruginosa by a LysR-Type Transcription Regulator
Source: PLoS Genet. 2009 Dec 18;5(12):e1000779. doi: 10.1371/journal.pgen.1000779 (PMC2796861; doi:10.1371/journal.pgen.1000779)
Supplement: Table S1 — Switching frequencies. (0.03 MB DOC) [file pgen.1000779.s006.doc]

| Strain Genotype | Transition | Switching Frequency (per cell generation, x10^-4^) |
| --- | --- | --- |
| *attB::P_bexR_-lacZ* | OFF → ON | 47.4 ± 6.6 |
|  | ON → OFF | 0.6 ± 0.4 |
| Δ*bexR* *attB::P_bexR_-lacZ* | OFF → ON | < 0.4 ± 0.1 |
